# Consumer Preferences for Low-Amylose Rice: A Sensory Evaluation and Best–Worst Scaling Approach

**DOI:** 10.3390/foods14122128

**Published:** 2025-06-18

**Authors:** Asato Mizuki, Hiroyuki Yasue

**Affiliations:** 1 Graduate School of Agricultural Science, Tohoku University, Sendai 980-8572, Japan; 2 NARO Development Strategy Center, National Agriculture and Food Research Organization, Tokyo 105-0003, Japan; yasue.hiroyuki571@naro.go.jp

**Keywords:** best–worst scaling, low-amylose rice, consumer evaluation, sensory test, food waste

## Abstract

This study investigates the influence of sensory evaluation results on consumer preference, specifically focusing on salted rice balls made from low-amylose rice, which is suitable for chilled rice applications. Sensory evaluations were conducted through home-use tests, and consumer behavior data were collected using the Best–Worst Scaling method. The results, analyzed via a conditional logit model, show that consumer preferences for new low-amylose rice varieties improved post-sensory evaluation, with stickiness and appearance exhibiting significant interaction effects. Although the preference for food waste reduction declined after the evaluation, positive responses remained consistently high both before and after the evaluation. The findings suggest that sensory characteristics may take precedence over other attributes in promoting processed rice products. Combining sensory evaluation with food experiences is crucial for understanding consumer preferences. Additionally, emphasizing the potential for shelf life extension and food loss reduction through low-amylose rice varieties can effectively raise consumer awareness.

## 1. Introduction

Our dietary habits have undergone significant changes over the past several decades. In recent years, traditional diets centered around staple foods have shifted toward more diversified eating patterns, characterized not only by an increased consumption of animal-based proteins such as meat and fish but also by a growing reliance on processed foods and eating out [1,2]. A similar trend has been observed in Japan, where traditional diets centered on plant-based foods and fish have shifted toward diets rich in animal products and dairy [3]. This phenomenon is often referred to as the Westernization of the Japanese diet [4]. With the Westernization of the Japanese diet, the annual per capita rice consumption has declined significantly—from 115 kg in 1960 to 51 kg in 2022 [5]. Correspondingly, rice production has also decreased, from 1214 tons in 1961 to 727 tons in 2022 [6]. Nevertheless, rice continues to provide approximately 22% of the total caloric intake in Japan, indicating that it remains the country’s staple food [5]. In addition to these trends, the “externalization of food” is progressing in developed countries [7,8,9,10]. This refers to the shift in food preparation and dining from home to outside establishments. This trend is also evident in Japan, where the proportion of food expenses spent on dining out and prepared foods increased from 13.4% in 1970 to 31.8% in 2024 [11]. Furthermore, the market size of prepared foods in Japan, including bento and deli foods that are cooked and processed outside the home and can be eaten without further cooking, was estimated at JPY 10.9 trillion in 2023, with expectations for continued market expansion [12]. Among these prepared foods, rice-based foods such as bento and onigiri comprise 43.9%, while general deli foods account for 34.9%. These foods are primarily sold at convenience stores (31.5%) and grocery supermarkets (29.7%).

In this context, there has been an increasing demand for chilled rice products within the prepared rice food category in Japan. Chilled rice, preserved under low-temperature refrigeration, offers improvements in shelf life and freshness compared to room temperature storage. Additionally, it allows for extended expiration dates, which is expected to significantly reduce food waste. This method aligns with sustainability goals and addresses growing consumer concerns about both food quality and environmental impact. Generally, refrigerated rice tends to harden, and its palatability deteriorates significantly due to the retrogradation of gelatinized starch, known as β-starch formation [13]. Starch, which makes up approximately 70% of brown rice, consists of amylose and amylopectin, with the amylose content playing a significant role in determining the texture and palatability of cooked rice [14]. The retrogradation process of rice is influenced by amylose and amylopectin, with varieties that have a higher amylose content retrograding more quickly [14]. Therefore, low-amylose rice has been recognized as a promising alternative due to its ability to preserve the taste and quality of freshly cooked rice, even after refrigeration [14]. Non-glutinous rice, widely consumed in Japan, has an amylose content of 17–23%, while low-amylose rice has an amylose content of 5–15%, positioning it between non-glutinous rice and glutinous rice [14]. Compared to regular non-glutinous rice, low-amylose rice remains sticky and soft after refrigeration, making it well-suited for chilled rice products [15]. However, traditional low-amylose rice varieties face challenges, such as low yields and operational losses due to rice grains sticking to forming machines during processing. Currently, research and development initiatives are focused on breeding high-yield rice varieties suitable for processing [16].

From a marketing perspective, promoting the adoption of low-amylose rice requires an understanding of its sensory characteristics and consumer preferences. Several studies have examined sensory evaluations of cooked rice [17,18,19,20]. Research using a Japanese consumer panel has shown that Japanese people prefer softer rice [21,22]. The sensory evaluation results of cooked low-amylose rice obtained by a Japanese panel revealed that, compared to Koshihikari, the most widely consumed Japonica rice in Japan, some low-amylose rice varieties have similar taste qualities. However, varieties with strong stickiness and a mochi-like odor are perceived as inferior in taste [23].

A choice experiment, a type of the stated preference method, is widely used to quantitatively evaluate consumer preferences for foods like low-amylose rice [24,25,26]. According to Aoki et al. [24], who studied consumer preferences for rice in Japan and Thailand, Japanese consumers show a high preference for organic rice but place lower priority on taste, with women tending to prefer premium rice. Mizuki and Yasue [27] analyzed consumer preferences for salted rice balls made with low-amylose rice and found that Japanese consumers positively evaluate low-amylose rice. The study also revealed that women and individuals with higher household incomes tend to prefer low-amylose rice.

Empirical studies on food waste reduction and consumer behavior have been conducted [28,29,30]. Ornelas et al. [29] found that consumers who actively reduce food waste tend to prefer circular food products. Additionally, Iwamoto [30] discovered that Japanese consumers positively evaluate companies engaged in food recycling. Ray et al. [31] combined a framework of consumer preferences for food waste and foods with varying expiration dates with a sensory evaluation of the foods, suggesting that expiration date labeling, along with sensory evaluations, may be effective in reducing food waste.

While extensive research has been conducted on sensory evaluation, consumer preferences for rice, and food waste reduction, studies specifically focused on low-amylose rice remain limited. Previous studies have primarily focused on general rice characteristics and consumer preferences related to food waste awareness and actions. However, insights into low-amylose rice remain sufficient. Bridging this gap is crucial not only for evaluating the added value of low-amylose rice but also for understanding its role in reducing food waste through processed rice meals. Therefore, this study aims to elucidate the influence of the sensory evaluation results on consumer preference, focusing on salted rice balls as a processed rice meal made from low-amylose rice. Unlike typical commercially available rice balls or bento boxes, salted rice balls contain no fillings and are seasoned only with salt, making them an ideal subject for evaluating the rice itself within processed rice meals.

## 2. Materials and Methods

### 2.1. Survey

In October 2022, we conducted a web survey designed specifically to gather responses to Case 3 Best–Worst Scaling (BWS) questions regarding consumer preferences for salted rice balls. Respondents for this survey were recruited from a large panel managed by MACROMILL, a major web research company in Japan. A total of 3293 individuals completed the screening survey. Of these, 59 participants who met the eligibility criteria for the test—specifically, those having consumed salted rice balls from a convenience store and owning a rice cooker with a capacity of two cups or more—were selected to take part in the survey. These participants resided in Tokyo and the neighboring prefectures of Saitama, Kanagawa, Chiba, and Ibaraki. In addition to selecting the best and worst attributes of salted rice balls made with low-amylose rice varieties, the survey asked respondents about their sex, age, income, and the frequency with which they purchase rice balls and salted rice balls at convenience stores.

**Figure 1:**
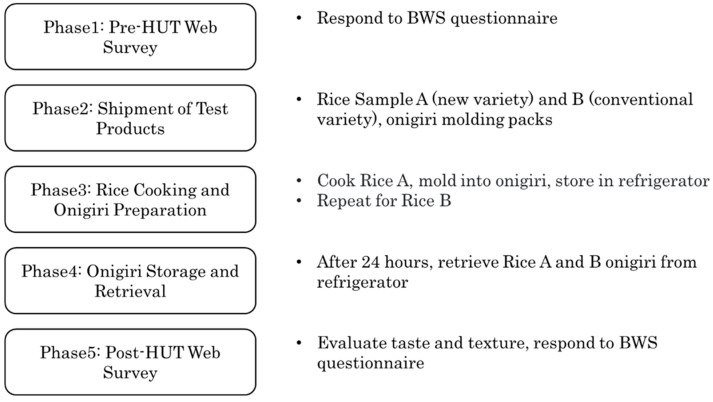
A diagram of the survey procedure.

Figure 1 illustrates the procedures for the web survey and the home-use test (HUT) conducted in this study. The BWS method was implemented twice: once before the taste evaluation and once after. The procedure involved five phases: (1) accessing the web survey and responding to the BWS questionnaire; (2) shipping two types of rice samples—Milled Rice A (new variety) and Milled Rice B (conventional variety)—along with two onigiri molding packs; (3) participants cooking Milled Rice A using their home rice cooker, molding the cooked rice into onigiri using the provided molding kit, storing it in the refrigerator, and repeating the procedure with Milled Rice B; (4) removing both Milled Rice A and Milled Rice B onigiri from the refrigerator 24 h after storing Milled Rice A onigiri; and (5) re-accessing the web survey to evaluate the taste and texture of the onigiri and responding to the BWS questionnaire. This approach allows us to understand how consumer evaluations change based on their experiences with taste testing.

### 2.2. Sensory Test

The sensory evaluation was conducted following the method outlined by the Food Agency [32]. Based on the Food Agency’s guidelines, six evaluation criteria for taste were established: appearance, aroma, taste, hardness, stickiness, and overall evaluation. For each of the six evaluation criteria, respondents assessed both the low-amylose and conventional non-glutinous rice varieties using a 5-point scale ranging from 1 to 5. As described above, participants received two types of polished rice at home, cooked them using their own rice cookers, stored the cooked rice in a refrigerator, and subsequently conducted a sensory evaluation. It is important to note that the evaluation was not conducted on the cooked rice itself, but by having respondents assess the rice formed into onigiri (rice ball) shapes. The statistical power of the test was 0.90 for a moderate effect size (f  =  0.3) for two groups with a significance level of 0.05 and a sample size of 59 [33].

### 2.3. Profile Design

To conduct an attribute evaluation of a hypothetical product, specifically salted rice balls made with low-amylose rice varieties, data regarding consumer attribute preferences were collected using Best–Worst Scaling (BWS). This method is categorized into three types: object case (Case 1), profile case (Case 2), and multi-profile case (Case 3) [34]. This study adopted a multi-profile case study to clarify the marginal willingness to pay (MWTP) for various attributes. The multi-profile BWS is considered an extension of the discrete choice experiment. While discrete choice experiments collect only the best responses, multi-profile BWS collects both the best and worst responses. This approach allows for the collection of more information from a single survey compared to discrete choice experiments. By utilizing multi-profile BWS, we aimed to gain deeper insights into consumer preferences and the perceived value of specific attributes related to salted rice balls made with low-amylose rice, offering a more comprehensive understanding of consumer behavior and potential market trends.

The attributes and levels used for the evaluation are shown in Table 1. Two levels of rice varieties were identified for comparison: the new low-amylose rice variety targeted in this study and a conventional variety that is not low in amylose. To reduce food loss, we considered a hypothetical food label indicating efforts to reduce food loss, with two levels representing the presence or absence of the label. Purchasing products with this label implies that consumers can contribute to food waste reduction. Since salted rice balls do not contain nori or fillings, the added value of the rice itself is considered a significant factor influencing consumer product choices. Therefore, the origin of the rice was included as an attribute, with two representative levels: Niigata Prefecture and domestically produced rice. In this survey, an orthogonal design was used to create the entire set of choice options, and each respondent answered eight BWS questions with different choice sets.

**Figure 2:**
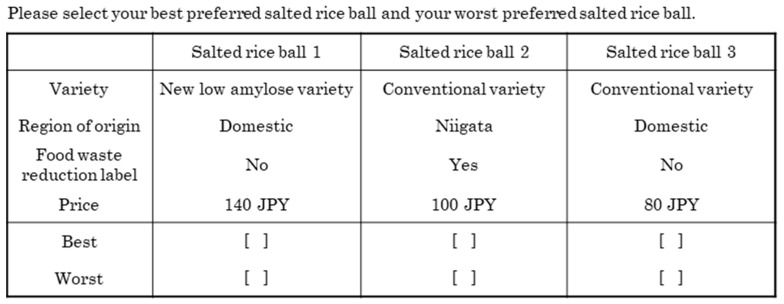
An example of a Case 3 Best–Worst Scaling question in the survey (translated from Japanese to English).

An example of a BWS question is shown in Figure 2. Given the potential differences in respondents’ knowledge of low-amylose rice varieties and food loss reduction efforts, the following information was provided before the BWS responses. The new low-amylose rice variety is characterized by its strong stickiness and ability to maintain its taste even when cooled. Additionally, 6.12 million tons of food waste occurs annually in Japan; convenience stores are developing products with longer shelf lives and encouraging the selection of items with approaching expiration dates. A “cheap talk” script was inserted before the BWS responses to reduce hypothetical bias in the stated preference survey.

### 2.4. Econometric Model

Our study employs a discrete choice modeling approach to understand consumer preferences for salted rice balls made from low-amylose rice varieties. This research aims to capture shifts in consumer preferences by utilizing two sets of choice data obtained from respondents: one collected before the taste evaluation and the other collected after. By analyzing these data, we can gain insights into how taste assessments influence consumer decisions and preferences regarding low-amylose rice products. The probability of choosing profile *ί* as the best and profile *ί′* as the worst in choice set *Χ* is given by

$$P_{BW}\left(ii^{\prime }|X\right)=\frac{exp\left(v_{i}-v_{i^{\prime }}\right)}{\sum_{\begin{array}{c} j,j^{\prime }\in X\\ j^{\prime }\neq j \end{array}}exp(v_{j}-v_{j^{\prime }})}$$

where $v_{i}$ is the component of the utility function [35].

We assume that a random utility function can be written as

$$U_{i}=v_{i}+\varepsilon =\beta_{variety}Variety+\beta_{region}Region+\beta_{foodwaste}Foodwaste+\beta_{price}Price+\varepsilon ,$$

where each β represents the utility parameter for each attribute to be estimated, and ε is a probability term. In addition, the willingness to pay can be obtained as follows:

$${WTP}_{i}=-\frac{\beta_{i}}{\beta_{price}}$$

The design and model estimation of the BWS were conducted using the support.BWS3, support.CEs, survival, and mlogit packages in R 4.1.2 (http://cran.r-project.org/).

## 3. Results and Discussion

### 3.1. Respondents’ Characteristics

Table 2 presents the socioeconomic characteristics of the respondents. As noted earlier, due to the dropout of one male participant in his twenties during the HUT, the sample has a slightly higher proportion of females compared to males. Furthermore, the proportion of respondents in their twenties is slightly lower. Regarding occupation, company employees make up the largest group at 44.1%, followed by full-time homemakers at 27.1%, and part-time workers or temporary workers at 18.6%. In terms of household income, the largest group, comprising 27.1% of respondents, falls within the income bracket of JPY 9 to 12 million. This is followed by 23.7% earning between JPY 3 and 6 million and 18.6% earning between JPY 6 and 9 million. Regarding family composition, 74.6% of respondents live with a spouse, which is the highest proportion, while 32.2% have children under the age of 18 living with them.

Table 3 shows the purchase frequency of rice balls and salted rice balls at convenience stores among the respondents. The percentage of respondents who purchase these items at least once a week is 49.2% for rice balls, compared to a lower 20.4% for salted rice balls. Notably, 55.9% of respondents purchase salted rice balls less than once a month, indicating that many respondents have less frequent opportunities to buy salted rice balls regularly.

### 3.2. Sensory Evaluation Results

Table 4 presents the results of a survey in which respondents were asked to rank the factors they consider most important when choosing rice. The data reveal that “taste” received the highest average rating of 4.75, indicating it is the most critical factor for respondents. This is followed by “aroma” with a score of 3.10 and “hardness” at 2.98. Conversely, “appearance” and “stickiness” were rated lower in importance, with scores of 2.17 and 2.00, respectively. Furthermore, an analysis of the standard deviation suggests greater variability in the ratings for “appearance” and “aroma”, indicating diverse opinions among respondents regarding these attributes. In contrast, the smaller standard deviation for “taste” implies a more consistent agreement on its importance, underscoring its central role in rice selection.

Table 5 presents the results of the sensory evaluation for salted rice balls conducted during the HUT. In this study, a sensory evaluation was conducted to compare the taste characteristics of salted rice balls prepared using Iwate 144, a low-amylose rice variety, and Koshihikari, a conventional non-glutinous rice variety. As shown in the table, the low-amylose rice received higher scores than the conventional non-glutinous rice across all six sensory evaluation categories, suggesting that salted rice balls prepared with low-amylose rice varieties possess superior palatability compared to those made with standard non-glutinous rice varieties. Notably, the items for overall evaluation, stickiness, and taste received particularly high scores. This can be interpreted as a reflection of the strong stickiness characteristic of the low-amylose rice variety, which retains its deliciousness even when cold, aligning with our expectations.

It is known that Japanese consumers tend to prefer cooked rice with a low amylose content, which results in a stickier texture [36]. However, low-amylose rice varieties developed in Japan in the past have been evaluated as stickier than Koshihikari but with a more pronounced “mochi” smell and slightly inferior taste [23]. The results of this study, however, show that the low-amylose rice varieties surpassed Koshihikari not only in stickiness but also in taste and overall evaluation. This can be attributed to the breeding goals of achieving both a high yield and an improved taste in the development of low-amylose rice varieties [16].

### 3.3. Estimation Results

Table 6 presents the results estimated using data from before and after the taste test. For the labels indicating Niigata Prefecture and food loss, the coefficient estimates were significant at the 1% level in both models, with positive signs. Although the new rice variety also had positive signs in both models, it was significant at the 1% level after the taste test compared to the 10% level before the taste test. Table 7 shows the point estimates of the MWTP and the 95% confidence intervals before and after the taste test, obtained using the results in Table 6. Before the taste test, the MWTP for Niigata Prefecture was the highest at 19.9 yen, followed by food loss labeling at JPY 15.4, with the new rice variety at the lowest value at JPY 5.9. However, after the taste test, the new rice variety became the highest at JPY 19.3, while food loss labeling and Niigata Prefecture were reversed to JPY 9.0 and JPY 8.3, respectively.

Table 8 presents the estimation results for the interaction effect model, which incorporates socioeconomic characteristic variables and taste evaluation variables as interaction terms into the main effect model described above. In the model before the taste test, which includes socioeconomic characteristic variables as interaction terms, the variables for Niigata Prefecture and female, Niigata Prefecture and the frequency of purchasing salted rice balls, and Niigata Prefecture and emphasis on taste were positive and significant. Additionally, the variables for food loss labeling and individuals in their 20s were negative and significant. In the model after the taste, which includes socioeconomic characteristic variables as interaction terms, the variables for the new rice variety and children were positive and significant, while the variables for the new rice variety and frequency of purchasing salted rice balls, as well as the new rice variety and individuals in their 20s, were negative and significant. Furthermore, in the model where taste evaluation variables were added as interaction terms, the variables for the new rice variety and appearance, and the new rice variety and stickiness, were positive and significant.

The results of the main effects model revealed that consumer preferences for low-amylose rice varieties improve after experiencing sensory evaluation. The attributes of goods, including food, are classified into search attributes and experience attributes based on their nature [38]. Search attributes are those that can be easily evaluated before purchase through exploration, while experience attributes are those that are difficult to evaluate before purchase and can only be assessed after consumption. In this study, information such as the origin and price of rice falls under search attributes, whereas sensory characteristics are considered experience attributes. It can be interpreted that the positive evaluation of the taste after consuming low-amylose rice, facilitated by the sensory evaluation, led to an increase in consumer preference for low-amylose rice post-sensory evaluation. The studies by Zapata et al. and Zhu et al. [39,40], which combined sensory evaluation with consumer preference surveys, suggest that sensory characteristics may be a factor that enhances consumer preference more than other search attributes, supporting the results of this study.

The indication for food waste reduction showed a positive willingness to pay both before and after sensory evaluation. While food waste reduction broadly possesses the attributes of a public good, consumers with strong altruism tend to have a higher willingness to pay for foods with public good attributes [41]. It is conceivable that the average respondent in this study might also exhibit strong altruism. Therefore, by conveying the message to consumers that the use of low-amylose rice varieties contributes to food waste reduction, it is possible to enhance the evaluation of processed rice meals made from low-amylose rice.

The results of the cross-effect model, which included sensory evaluation variables, indicated the existence of interaction effects between low-amylose rice varieties and the variables of appearance and stickiness. Stickiness received a high evaluation, second only to the overall evaluation in the sensory assessment results shown in Table 5, suggesting that respondents positively assessed the strength of stickiness in low-amylose rice varieties. In contrast, the appearance was relatively lower in the sensory evaluation results, and the factors contributing to this outcome are not clearly understood.

## 4. Conclusions

This study analyzed the influence of the sensory evaluation results on consumer preference, focusing on salted rice balls made from low-amylose rice suitable for chilled rice applications. An HUT was conducted to evaluate the sensory characteristics of low-amylose rice, and consumer selection behavior data for salted rice balls was collected using the BWS method. Consumer preferences were estimated using a conditional logit model. The estimation results reveal that consumer preferences for new varieties of low-amylose rice improve after sensory evaluation. Among sensory characteristics, stickiness and appearance were found to have interaction effects with low-amylose rice. Although consumer preferences for food waste reduction decreased after sensory evaluation, positive evaluations were obtained both before and after the sensory evaluation.

The analysis of consumer preferences combined with sensory evaluation suggests that sensory characteristics may take priority over other attributes in the promotion of processed rice products made from new low-amylose rice varieties. In promoting new food products like those targeted in this study, combining sensory evaluation with food experience can provide a better understanding of consumer preferences. Furthermore, given the high evaluation of consumers for food waste reduction, raising awareness about the potential for extending shelf-life and contributing to food loss reduction through the use of low-amylose rice varieties can be considered effective.

One limitation of this study is the limited sample of respondents. Respondents were drawn from the metropolitan area centered around Tokyo, Japan, and the sample size was restricted to 59 participants due to the sensory evaluation conducted through HUTs. Therefore, further verification is needed to determine whether the results of this study are representative of the broader Japanese consumer population.

## Figures and Tables

**Table 1:** Attribute and level.

Attribute	Level
Variety	New low-amylose variety, conventional variety
Region of origin	Niigata, domestic
Food waste reduction label (yes: attached)	Yes, no
Price	JPY 80, JPY 100, JPY 120, JPY 140

**Table 2:** Characteristics of respondents.

	%
Sex	
Male	49.2
Female	50.8
Age (years)	
20–29	18.6
30–39	20.3
40–49	20.3
50–59	20.3
60–69	20.3
Employment	
Office workers	44.1
Part-time workers	18.6
Housewives	27.1
Students	5.1
Others	5.1
Household income (million JPY per year)	
Less than 3	5.1
3–6	23.7
6–9	18.6
9–12	27.1
Over 12	8.5
No response	16.9
Family members living together (multiple responses)	
Partners	74.6
Children under 18 years	32.2
Children over 18 years	18.6
Parents of brothers	17.0
No family members	10.2

Note: Office workers are generally employed on a full-time basis, receiving benefits and stable salaries. Conversely, part-time workers engage in shorter or irregular work hours and are typically compensated on an hourly basis, with limited access to employment benefits.

**Table 3:** Purchase frequency of rice balls and salted rice balls.

	Rice Balls	Salted Rice Balls
Three times or more per week	15.3	8.5
Once or twice per week	33.9	11.9
Two or three times per month	49.2	23.7
Less than once per month	1.7	55.9
Do not purchase	0.0	0.0

Note: Data are presented as n (%) otherwise indicated.

**Table 4:** Important factors for rice.

	Mean	Standard Deviation
Appearance	2.17	1.29
Aroma	3.10	1.21
Taste	4.75	0.54
Hardness	2.98	1.01
Stickiness	2.00	0.95

Note: The five items were ranked in order of importance from 1st to 5th place, with the 1st place assigned a score of 5 and the 5th place assigned a score of 1 for quantification.

**Table 5:** Sensory evaluation of salted rice balls.

	Means		*p*-Value
	Non-Glutinous Rice Koshihikari	Low Amylose-Rice Iwate 144	
Appearance	3.31	3.68	0.007
Aroma	3.34	3.80	0.002
Taste	3.37	3.86	0.000
Hardness	3.47	3.83	0.018
Stickiness	3.36	3.93	0.000
Overall Evaluation	3.37	3.97	0.000

**Table 6:** Estimation results.

	Before Tasting			After Tasting		
Variables	Coefficient	Standard Error	z-Value	Coefficient	Standard Error	z-Value
New variety	0.125 *	0.067	1.870	0.475 ***	1.608	6.839
Niigata	0.439 ***	0.068	6.457	0.204 ***	1.227	2.973
Food waste	0.338 ***	0.067	5.020	0.222 ***	1.248	3.235
Price	−0.023 ***	0.002	−13.697	−0.025 ***	0.976	−14.354
Respondents	59			59		
Observations	472			472		
Log likelihood at zero	−845.71			−845.71		
Log likelihood at convergence	−695.52			−675.89		
Adjusted pseudo R^2^	0.173			0.196		

*** *p* < 0.01, * *p* < 0.1.

**Table 7:** MWTP estimation (before and after tasting).

	Before Tasting		After Tasting		Change
New variety	5.86	[−0.31, 12.24]	19.30	[13.80, 25.16]	13.44
Niigata	19.93	[13.66, 26.77]	8.30	[2.84, 13.95]	−11.63
Food Waste	15.43	[9.42, 22.10]	9.01	[3.59, 14.80]	−6.42

Note: The 95% confidence intervals in the brackets are derived by 10,000 replications using the method introduced by Krisnky and Robb [37].

**Table 8:** Estimation results (cross effect).

	Before Tasting			After Tasting			After Tasting		
Variables	Coefficient	Standard Error	z-Value	Coefficient	S.E.	z-Value	Coefficient	Standard Error	z-Value
Main Effect									
New variety	0.451 **	0.210	0.032	0.951 ***	0.220	0.000	0.633 **	0.253	0.012
Niigata	−0.241	0.210	0.252	0.113	0.219	0.607	0.108	0.220	0.623
Food waste	0.616 ***	0.211	0.004	0.410 *	0.219	0.062	0.416 *	0.220	0.058
Price	−0.023 ***	0.002	0.000	−0.025 ***	0.002	0.000	−0.026 ***	0.002	0.000
Cross Effect									
New variety × female	−0.027	0.148	0.853	0.055	0.153	0.718	0.023	0.159	0.885
Niigata × female	0.464 ***	0.149	0.001	0.145	0.153	0.343	0.157	0.157	0.316
Food waste × female	−0.092	0.149	0.538	0.097	0.153	0.525	0.106	0.157	0.498
New variety × fsrb	−0.099	0.080	0.221	−0.022 ***	0.082	0.005	−0.148*	0.089	0.096
Niigata × fsrb	0.157*	0.080	0.051	0.061	0.081	0.452	0.070	0.085	0.405
Food waste × fsrb	−0.031	0.081	0.704	−0.058	0.082	0.475	−0.058	0.085	0.491
New variety × 20s	−0.066	0.178	0.712	−0.509 ***	0.181	0.005	−0.279	0.195	0.151
Niigata × 20s	−0.111	0.177	0.531	0.062	0.179	0.726	0.063	0.183	0.730
Food waste × 20s	−0.310*	0.178	0.083	−0.133	0.181	0.462	−0.143	0.184	0.436
New variety × child	0.141	0.156	0.370	0.393 **	0.163	0.016	0.383 **	0.177	0.030
Niigata × child	−0.090	0.156	0.565	−0.069	0.163	0.669	−0.076	0.165	0.642
Food waste × child	−0.198	0.157	0.206	−0.244	0.163	0.134	−0.256	0.166	0.122
New variety × taste	−0.212	0.174	0.223	−0.146	0.183	0.424	−0.163	0.194	0.401
Niigata × taste	0.288*	0.174	0.098	−0.085	0.183	0.640	−0.095	0.187	0.608
Food waste × taste	−0.056	0.174	0.746	−0.018	0.183	0.921	−0.009	0.186	0.957
New variety × appearance							0.300 ***	0.084	0.000
New variety × aroma							−0.096	0.095	0.312
New variety × taste							0.048	0.093	0.601
New variety × hardness							0.012	0.085	0.884
New variety × stickiness							0.167*	0.091	0.067
Respondents	59			59			59		
Observations	472			472			472		
Log likelihood at zero	−845.711			−845.711			−845.711		
Log likelihood at convergence	−680.068			−657.692			−646.081		
Adjusted pseudo R^2^	0.169			0.199			0.207		

*** *p* < 0.01, ** *p* < 0.05, * *p* < 0.1.

## Data Availability

The original contributions presented in this study are included in the article. Further inquiries can be directed to the corresponding author.
